# The CTLA-4 rs231775 GG genotype is associated with favorable 90-day survival in Caucasian patients with sepsis

**DOI:** 10.1038/s41598-018-33246-9

**Published:** 2018-10-11

**Authors:** Caspar Mewes, Benedikt Büttner, José Hinz, Ayelet Alpert, Aron Frederik Popov, Michael Ghadimi, Tim Beissbarth, Mladen Tzvetkov, Shai Shen-Orr, Ingo Bergmann, Ashham Mansur

**Affiliations:** 1 Department of Anesthesiology, University Medical Center, Georg August University, Robert-Koch-Str. 40, D-37075 Goettingen, Germany; 2 OP-Management, University Medical Center, Georg August University, Robert-Koch-Str. 40, D-37075 Goettingen, Germany; 3 Faculty of Medicine, Technion–Israel Institute of Technology, Haifa, Israel; 4 Department of Thoracic and Cardiovascular Surgery, University Medical Center, Eberhard Karls University, Hoppe-Seyler-Straße 3, D-72076 Tuebingen, Germany; 5 Department of General and Visceral Surgery, University Medical Center, Georg August University, Robert-Koch-Str. 40, D-37075 Goettingen, Germany; 6 Department of Medical Statistics, University Medical Center, Georg August University, Robert-Koch-Str. 40, D-37075 Goettingen, Germany; 7 Department of Clinical Pharmacology, University Medical Center, Georg August University, Robert-Koch-Str. 40, D-37075 Goettingen, Germany; 8 Department of Clinical Pharmacology, University Medical Center, Ernst-Moritz-Arndt-University, Felix-Hausdorff-Str. 3, D-17487 Greifswald, Germany

## Abstract

Cytotoxic T-lymphocyte-associated protein 4 (CTLA-4) is a surface protein on T cells, that has an inhibitory effect on the host immune reaction and prevents overreaction of the immune system. Because the functional single-nucleotide polymorphism (SNP) rs231775 of the CTLA-4 gene is associated with autoimmune diseases and because of the critical role of the immune reaction in sepsis, we intended to examine the effect of this polymorphism on survival in patients with sepsis. 644 septic adult Caucasian patients were prospectively enrolled in this study. Patients were followed up for 90 days. Mortality risk within this period was defined as primary outcome parameter. Kaplan-Meier survival analysis revealed a significantly lower 90-day mortality risk among GG homozygous patients (n = 101) than among A allele carriers (n = 543; 22% and 32%, respectively; p = 0.03565). Furthermore, the CTLA-4 rs231775 GG genotype remained a significant covariate for 90-day mortality risk after controlling for confounders in the multivariate Cox regression analysis (hazard ratio: 0.624; 95% CI: 0.399–0.975; p = 0.03858). In conclusion, our study provides the first evidence for CTLA-4 rs231775 as a prognostic variable for the survival of patients with sepsis and emphasizes the need for further research to reveal potential functional associations between CTLA-4 and the immune pathophysiology of sepsis.

## Introduction

Sepsis is defined as a “life-threatening organ dysfunction caused by a dysregulated host response to infection^1^. It is a significant public health care issue^2^ and still a leading cause of death and critical illness in intensive care units (ICUs) worldwide^3^. Studies suggest a number of 31.5 million sepsis cases and 5.3 million potential sepsis-related deaths annually worldwide, extrapolated from high-income country data^4^. Despite substantial progress in the clinical understanding of sepsis, the evolution of the definition, advanced antibiotic therapies, ventilation and fluid management^5,6^, a sepsis-specific treatment is nonexistent^7^. Considering the key role of the immune pathophysiology of sepsis, immune therapy seems to be an attractive target in the treatment of sepsis^8^.

The immune response in sepsis can be characterized by two merging phases: an initial cytokine-mediated hyperinflammatory phase with an inappropriately amplified systemic immune reaction followed by an anti-inflammatory state of immune suppression^9^. While prior clinical investigations and trials focused on suppressing the initial hyperinflammatory phase^10^, it is now clear that most patients survive this phase of the disease and die instead during the subsequent phase of immune suppression^11,12^. This phase is characterized by dysfunction of the innate and adaptive immune system leading to increased host vulnerability to secondary bacterial infections, multiple organ dysfunction and the reactivation of latent viruses such as cytomegalovirus (CMV) or herpes simplex virus (HSV)^10–13^. A variety of mechanisms is considered to be responsible for the sepsis-induced immune suppression, including an increase in regulatory T cells, cellular exhaustion and apoptotic depletion of immune cells^9^. Furthermore, upregulated expression of certain cell surface receptors on hematopoietic cells such as coinhibitory receptors programmed cell death 1 protein (PD-1) or CTLA-4 (i.e., checkpoint proteins) has been reported^14,15^.

PD-1 and CTLA-4 are suggested to play a key role in the host response over the course of sepsis^16,17^; these proteins function as concomitant inhibitory receptors within the process of T cell activation and proliferation^18^. While antigen-presenting cells (APC), such as macrophages, dendritic cells or monocytes, present antigens from foreign pathogenic sources on MHC II towards T cell receptor-expressing lymphocytes, costimulatory and coinhibitory cascades occur simultaneously (e.g., costimulatory receptors CD40 and CD28; coinhibitory receptors PD-1 and CTLA-4)^19^. In particular, CTLA-4 is a T cell surface protein that competes with CD28 for binding to CD80 and CD86 on APCs^19,20^. Thus, overexpression of CTLA-4 downregulates the activation and proliferation of T cells and therefore has an inhibitory effect on the host immune reaction, preventing an overreaction of the immune system^21,22^. CTLA-4 is reported to play a role in sepsis-induced immune suppression and the development of septic morbidity^19^ and may be important in the exploration of future therapy targets. While CTLA-4-specific monoclonal antibodies are already being used in therapy for malignant melanoma and prostate cancer^23,24^, mouse models showed similar positive effects in the treatment of sepsis^25,26^.

Genetic variations within the CTLA-4 gene, which is located on chromosome 2q33, may cause an attenuated inhibitory effect on the T cell reaction^27^. We therefore examined the functional SNP rs231775 in exon 1 of the CTLA-4 gene, which is reported to be associated with several systemic and autoimmune diseases such as insulin-dependent diabetes mellitus (IDDM)^28^, rheumatoid arthritis and Hashimoto thyroiditis^29^. The rs231775 SNP (also referred to as CTLA-4 +49 A/G polymorphism) causes a substitution of threonine (Thr) to alanine (Ala) in the CTLA-4 receptor, and guanine at this position is related to lower expression levels of the CTLA-4 protein^30^. Furthermore, studies have reported that the rs231775 GG genotype is associated with higher T cell activation and proliferation and is, as previously stated, more frequent in Caucasian patients with the autoimmune diseases rheumatoid arthritis and Hashimoto thyroiditis^29^.

Our study aimed to investigate whether mortality among patients with sepsis was associated with the CTLA-4 rs231775 polymorphism. The 90-day mortality risk was recorded as the primary outcome parameter. We hypothesized that the CTLA-4 rs231775 GG genotype is beneficial in the course of sepsis due to the previously mentioned lower expression levels of the CTLA-4 protein, resulting in higher activation and proliferation of T cells in the immunosuppressive stage of sepsis.

## Results

### Baseline characteristics

A cohort of 644 adult Caucasian patients was enrolled in this study. On average, the patients were 63 years old (range: 18–92; standard deviation: ±15; median: 65) [Table 1]; 66% of the patients were male individuals, while 34% were female. Upon enrollment, the cohort’s mean Sequential Organ Failure Assessment (SOFA) score was 9.4 ± 3.9, and the Acute Physiology and Chronic Health Evaluation (APACHE II) was measured at 22 ± 7. Of the 644 patients, 86% were mechanically ventilated, 67% received vasopressors, and 9% obtained renal replacement therapy [Table 1]. During the observation period, 51% of the patients were in septic shock. The genotype distribution of CTLA-4 rs231775 was 101:289:254 (GG:AG:AA) with an observed minor allele frequency (MAF) of 0.38. Consequently, the genotype distribution is consistent with Hardy-Weinberg equilibrium (p = 0.2156), and the observed MAF almost equals the expected HapMap CEPH (CEU) MAF of 0.39^31^. In accordance with our a priori hypothesis, we pooled the CTLA-4 rs231775 AA and AG genotypes in order to compare the clinical course of GG homozygotes with the A allele carriers.

**Table 1:** Baseline characteristics with regard to CTLA-4 rs231775 genotypes.

Parameter	All (n = 644)	rs231775		p value
		GG (n = 101)	AA/AG (n = 543)	
Age, years	63 ± 15	63 ± 14	63 ± 15	0.5354
Male [%]	66	62	67	0.3829
Body mass index (BMI)	28 ± 6	29 ± 7	27 ± 6	0.0365
**Severity of sepsis**				
Septic shock, %	51	50	52	0.6786
Sequential Organ Failure Assessment (SOFA) score	9.4 ± 3.9	9.8 ± 3.9	9.3 ± 3.9	0.3057
Acute Physiology and Chronic Health Evaluation (APACHE II) score	22 ± 7	22 ± 7	22 ± 7	0.5020
**Comorbidities, n [%]**				
Hypertension	54	54	54	0.9540
History of myocardial infarction	5	6	2	0.0954
Chronic obstructive pulmonary disease	15	15	15	0.9486
Renal dysfunction	10	11	10	0.8613
Noninsulin-dependent diabetes mellitus	9	11	8	0.4318
Insulin-dependent diabetes mellitus	11	14	10	0.2396
Chronic liver disease	6	11	6	0.0425
History of cancer	16	13	16	0.4221
History of stroke	6	6	6	0.9852
**Recent surgical history, n [%]**				
Elective surgery	29	25	30	
Emergency surgery	53	57	52	
No history of surgery	18	18	18	
**Site of infection, n [%]**				
Lung	62	63	62	
Abdomen	20	22	20	
Bone or soft tissue	4	4	4	
Surgical wound	2	2	1	
Urogenital	2	1	3	
Primary bacteremia	7	3	7	
Other	3	5	3	
**Organ support [%]**				
Used during observation period				
Mechanical ventilation	93	95	93	0.4491
Use of vasopressor	79	81	79	0.5906
Renal replacement therapy	21	27	20	0.1102
Used on sepsis onset				
Mechanical ventilation	86	88	85	0.4520
Use of vasopressor	67	70	66	0.3928
Renal replacement therapy	9	14	8	0.0535
Use of statins [%]	24	23	24	0.7999
ICU length of stay	20 ± 16	19 ± 12	21 ± 17	0.5142

### Outcomes

**Figure 1:**
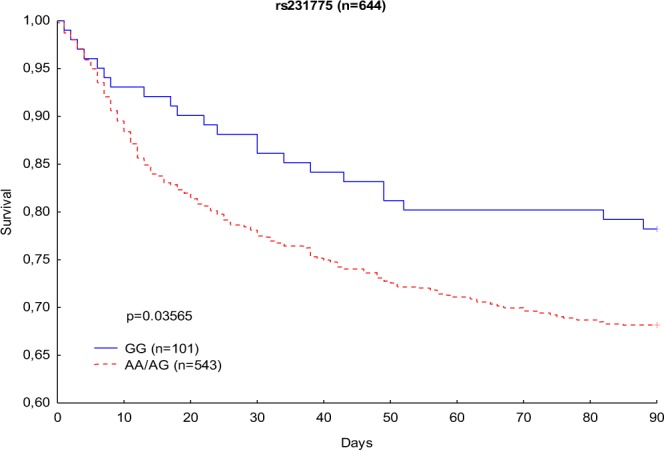
Kaplan-Meier survival analysis (90 days) with regard to CTLA-4 rs231775 genotypes.

The Kaplan-Meier analysis of 90-day survival revealed a significantly lower 90-day mortality risk among GG homozygous patients than among A allele carriers (22% and 32%, respectively; p = 0.03565) [Fig. 1].

**Figure 2:**
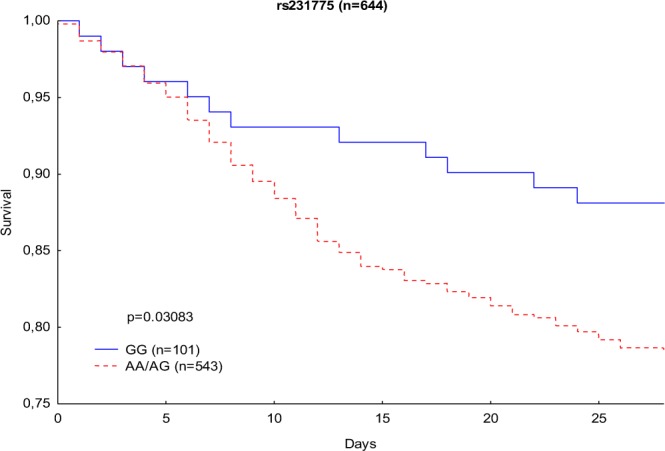
Kaplan-Meier survival analysis (28 days) with regard to CTLA-4 rs231775 genotypes.

In addition, 28-day mortality risk was examined using the Kaplan-Meier survival analysis and showed similar effects; GG homozygous patients had a lower 28-day mortality risk than A allele carriers (13% and 22%, respectively; p = 0.03083) [Fig. 2].

The analysis of the baseline characteristics revealed a significantly higher average body mass index (BMI) among GG genotypes (29 ± 7) than that among A allele carriers (27 ± 6; p = 0.0365) and, likewise, a higher percentage of chronic liver disease as a comorbidity (11% vs. 6%; p = 0.0425) [Table 1].

To adjust for possible effects of different baseline characteristics and potential confounders on the 90-day mortality, a multivariate Cox regression analysis was performed. Age, male gender, and SOFA and APACHE II scores were included as baseline variables; potential confounder variables were defined as “no statin therapy” in accordance with our findings from previous studies^32^ as well as BMI and chronic liver disease based on the results of the baseline characteristics. As patients were enrolled into the study according to the at that time of enrollment valid sepsis definition (Sepsis-1 or Sepsis-3) “application of Sepsis-3 definition” was also included in the potential confounder variables. The GG genotype remained a significant covariate for 90-day mortality risk (hazard ratio: 0.624; 95% CI: 0.399-0,975; p = 0.03858) [Table 2], indicating that despite potential confounders, the CTLA-4 rs231775 GG genotype is an independent prognostic variable for survival of patients with sepsis. Cox regression analysis for 28-day mortality revealed similar results for the CTLA-4 rs231775 GG genotype (hazard ratio: 0.504; 95% CI: 0.277–0.917; p = 0.02487) [Table 3].

**Table 2:** Cox regression analysis (90 days) with regard to CTLA-4 rs231775 genotypes.

Variable	Hazard ratio	95% CI	p value
Age	1.027	1.015–1.040	0.000011
Male gender	1.091	0.807–1.476	0.569877
Body mass index (BMI)	0.981	0.957–1.008	0.164128
Sequential Organ Failure Assessment (SOFA) score	1.076	1.029–1.126	0.001499
Acute Physiology and Chronic Health Evaluation (APACHE II) score	1.034	1.005–1.063	0.019803
No statin therapy	1.057	0.757–1.475	0.744880
Chronic liver disease	0.881	0.476–1.631	0.686610
Application of Sepsis-3 definition	0.760	0.452–1.279	0.301009
GG genotype	0.624	0.399–0.975	0.038577

**Table 3:** Cox regression analysis (28 days) with regard to CTLA-4 rs231775 genotypes.

Variable	Hazard ratio	95% CI	p value
Age	1.025	1.011–1.040	0.000694
Male gender	1.278	0.872–1.874	0.208204
Body mass index (BMI)	0.979	0.948–1.011	0.203222
Sequential Organ Failure Assessment (SOFA) score	1.089	1.030–1.152	0.002579
Acute Physiology and Chronic Health Evaluation (APACHE II) score	1.034	0.999–1.069	0.056276
No statin therapy	1.234	0.809–1.881	0.328568
Chronic liver disease	0.982	0.476–2.028	0.961675
Application of Sepsis-3 definition	0.844	0.460–1.549	0.583507
GG genotype	0.504	0.277–0.917	0.024871

Accordingly, male gender, BMI, “no statin therapy”, chronic liver disease and “application of Sepsis-3 definition” had no significant effect on mortality in the multivariate Cox regression analysis [Tables 2 and 3]. Further analysis can be found in the supplementary information file.

### Disease severity

CTLA-4 rs231775 genotypes have also been evaluated regarding disease severity, the requirement of organ support, and inflammatory, kidney and liver parameters [Table 4]. Average SOFA and organ-specific SOFA scores from the first 28 days after sepsis onset (unless previously dismissed or deceased) were compared between the CTLA-4 rs231775 genotypes. No significant differences were found. As a measure of organ dysfunction, we assessed organ support-free days and days of ventilation, vasopressor requirement and dialysis as a fraction of the observation days. Similarly, these indicators did not differ between the CTLA-4 rs231775 genotype groups.

**Table 4:** Disease severity with regard to CTLA-4 rs231775 genotypes.

Variable	All (n = 644)	rs231775		p value
		GG (n = 101)	AA/AG (n = 543)	
SOFA	7.0 ± 3.5	7.1 ± 3.4	7.0 ± 3.6	0.5009
SOFA-Respiratory score	2.0 ± 0.8	2.0 ± 0.7	2.0 ± 0.8	0.9854
SOFA-Cardiovascular score	1.5 ± 1.0	1.5 ± 1.0	1.5 ± 1.0	0.9986
SOFA-Central nervous system score	2.0 ± 1.1	2.0 ± 1.1	2.0 ± 1.1	0.9883
SOFA-Renal score	0.8 ± 1.2	0.9 ± 1.3	0.7 ± 1.1	0.6040
SOFA-Coagulation score	0.4 ± 0.6	0.3 ± 0.6	0.4 ± 0.6	0.4978
SOFA-Hepatic score	0.4 ± 0.7	0.4 ± 0.8	0.4 ± 0.7	0.6419
Length of stay in ICU [days]	20 ± 16	19 ± 12	21 ± 17	0.5142
**Organ support-free days**				
Ventilator-free days	5 ± 5	5 ± 5	5 ± 5	0.1947
Vasopressor-free days	10 ± 7	10 ± 6	10 ± 7	0.4826
Dialysis-free days	14 ± 8	13 ± 7	14 ± 8	0.1556
Ventilation days/observation days, [%]	66 ± 32	64 ± 29	67 ± 32	0.1969
Vasopressor days/observation days, [%]	34 ± 30	34 ± 30	34 ± 30	0.7022
Dialysis days/observation days, [%]	9 ± 23	12 ± 23	9 ± 23	0.2690
**Inflammatory values**				
Leukocytes [1000/µl]	13 ± 5	13 ± 4	13 ± 5	0.8124
CRP [mg/l]	152 ± 87	177 ± 113	148 ± 81	0.2758
Procalcitonin [ng/dl]	4.0 ± 9.3	5.2 ± 10.3	3.8 ± 9.1	0.1124
**Kidney values**				
Urine output [ml/day]	2978 ± 1337	2806 ± 1179	3010 ± 1363	0.2993
Urine output [ml/kg/day]	1.6 ± 0.8	1.4 ± 0.8	1.6 ± 0.8	0.1014
Creatinine [mg/dl]	1.2 ± 0.9	1.3 ± 1.1	1.2 ± 0.9	0.3945
**Liver values**				
AST (GOT) [IU/l]	169 ± 598	162 ± 329	170 ± 630	0.2059
ALT (GPT) [IU/l]	94 ± 188	90 ± 174	94 ± 191	0.5080
Bilirubin [mg/dl]	1.2 ± 2.1	1.4 ± 3.0	1.2 ± 1.8	0.8423

Our analysis of microbiological findings [Table 5] indicated some interesting tendencies: common pathogens such as *Staphylococcus epidermidis*, *Candida albicans* or *Pseudomonas aeruginosa* were microbiologically found less often in GG homozygous patients than in A allele carriers in our cohort, while *Staphylococcus aureus* was found more often. These findings are, however, not significant and may also be further investigated in other studies or larger cohorts. Further analysis can be found in the supplementary information file.

**Table 5:** Microbiological findings according to CTLA-4 rs231775 genotypes.

Variable	All (n = 644)	rs231775		p value
		GG (n = 101)	AA/AG (n = 543)	
**Total infections [%]**				
Gram-positive infection	78	83	78	0.2062
Gram-negative infection	67	70	67	0.4538
Fungal infection	52	49	52	0.4633
Viral infection	13	14	12	0.6718
**Bronchial infections [%]**				
Gram-positive infection	42	49	41	0.1439
Gram-negative infection	45	50	45	0.3600
Fungal infection	33	28	34	0.2007
Viral infection	2	0	2	0.1491
**Blood culture findings [%]**				
Gram-positive finding	21	19	21	0.5630
Gram-negative finding	5	3	6	0.2584
Fungal finding	12	6	13	0.0626
Viral finding	11	14	11	0.3832
Positive blood culture of any type	40	39	40	0.7992
**Abdominal findings [%]**				
Gram-positive finding	15	13	16	0.4747
Gram-negative finding	11	11	11	0.9940
Fungal finding	10	7	10	0.2934
**Catheter infections [%]**				
Gram-positive infection	19	20	19	0.9136
Gram-negative infection	6	6	6	0.9578
**Pathogen findings [%]**				
*Staphylococcus epidermidis*	34	30	35	0.3373
*Candida albicans*	30	27	31	0.3982
*Escherichia coli*	28	28	28	0.8959
*Staphylococcus aureus*	22	27	22	0.2508
Enterococcus species	20	20	20	0.9165
*Enterococcus faecalis*	20	18	20	0.6305
*Enterococcus faecium*	16	14	16	0.6145
*Pseudomonas aeruginosa*	13	14	8	0.0879
*Candida glabrata*	11	12	11	0.7648
*Klebsiella pneumoniae*	10	8	11	0.3734
*Proteus mirabilis*	9	7	10	0.3690

## Discussion

Considering the complexity of the immune response in sepsis, it is important to examine factors involved in the activation and upregulation of the immune system, such as the coinhibitory checkpoint protein CTLA-4. Although SNPs are not known to be direct causative factors of diseases, their impact on gene expression and its products makes them important factors in disease susceptibility^33^.

The present study aimed to assess a potential association between the CTLA-4 rs231775 SNP and the survival of Caucasian patients with sepsis. We defined 90-day mortality as a primary outcome parameter. The main finding of our study was that CTLA-4 rs231775 GG homozygous patients have a significantly better 90-day mortality risk than A allele carriers. To the best of our knowledge, this study is the first investigation evaluating the effect of CTLA-4 rs231775 on mortality among patients with sepsis. Our findings are consistent with previous studies showing an association between CTLA-4 rs231775 genotypes and systemic or autoimmune diseases^28,29^ as well as recent mouse models with improved survival of fungal sepsis after anti-CTLA-4 treatment^26^. A potential explanation for the detected independent strong impact of the CTLA-4 rs231775 GG genotype on mortality risk is that the previously described reduced cell surface expression of coinhibitory CTLA-4^30^ and the consequent increase in T cell activation and proliferation^34^ in GG homozygous patients might be beneficial during the immunosuppressive state of sepsis. In other words, the GG genotype most likely has a reduced inhibitory function of CTLA-4, resulting in a less pronounced sepsis-associated immunosuppression and thus an enhanced clinical course, especially in the late phase of sepsis. Similarly, the observed lower 28-day mortality risk among CTLA-4 GG patients may be due to the less distinctive immunosuppressive component exhibited by these patients within 28 days after sepsis onset. However, these assumed connections need to be further investigated and future genetic and functional studies need to precisely reveal the biological mechanisms underlying our findings.

As secondary endpoints, our cohort showed no significant genotype-based differences in disease severity, required organ support as a measure of organ dysfunction or bacterial findings. This observation indicates that the parameters used may not be able to adequately represent sepsis-associated immunosuppression, especially in the late phase of sepsis. Patients with the CTLA-4 rs231775 GG genotype had significantly higher average BMI values and a higher occurrence of chronic liver disease than patients carrying the A allele. However, no significant effect on survival was detected in the multivariate Cox regression for these two parameters, so they can be neglected as confounders in the association between CTLA-4 rs231775 genotypes and survival. Nevertheless, our finding of differences in BMI and the occurrence of chronic liver disease requires verification in other cohorts to validate its clinical relevance.

As far as our study is a genetic association study, potential limitations such as inadequate sample size, multiple testing and population stratification should be considered. Regarding these concerns, a relatively large population of septic patients and a reduced selection bias due to the prospective cohort design can certainly be seen as strengths of our study^35^. However, the inclusion of Caucasian only septic patients is a limitation of the present study as results might not be generalizable for other ethnicities. It would be interesting to assess the observed effects in cohorts of other ethnicity to allow generalization of the clinical relevance of our investigations. Similarly, our cohort consists of severely ill surgical patients, so that some of our observations may not be representative for other ICUs (e.g. medical ICU). Additionally, a power calculation to determine an adequate sample size could not be conducted at the beginning of our study because of the initially unknown effect of CTLA-4 rs231775 on the outcome of septic patients. Though, a post hoc power analysis of the observed mortality rates and our sample size was conducted and showed a power of 0.98. Furthermore, our study is a single-center study, and the results need to be validated in other independent cohorts.

In conclusion, our study provides the first evidence for CTLA-4 rs231775 as a prognostic variable for the survival of Caucasian patients with sepsis. Our findings may help identify patients at risk in clinical settings and emphasize the need for further research to reveal potential functional associations between CTLA-4 and the immune pathophysiology of sepsis. Our study also suggests using CTLA-4 gene variants to stratify patients for anti-CTLA-4 therapy.

## Methods

### Patients

A total of 644 septic patients were enrolled in this study through the GENOSEP database of the Department of Anesthesiology at the University Medical Center Goettingen. The patients were prospectively recruited from three surgical ICUs since March 2012. All patients of these ICUs were screened for sepsis according to the actual guidelines and definitions^1,36^ on a daily basis. The majority of 591 patients was enrolled into the study according to the definitions for sepsis and organ failure by the American College of Chest Physicians and Society of Critical Care Medicine Consensus Conference Committee (Sepsis-1)^36^. After the publication of the third international consensus definitions for sepsis and septic shock (Sepsis-3)^1^ in February 2016 another 53 patients were enrolled into the study according to the new guidelines. Blood was drawn from all patients within 72 hours of sepsis onset, and patients were followed up for 90 days unless previously dismissed from the ICU or deceased. For homogeneity reasons, only Caucasian patients were included in the study, and the following previously described exclusion criteria were applied^17^: (1) immunosuppressive therapy (e.g. azathioprine, cyclosporine, glucocorticoids) and cancer-related chemotherapy or (2) myocardial infarction within six weeks before enrollment, (3) human immunodeficiency virus (HIV) infection, (4) congestive heart failure as classified by the New York Heart Association (NYHA) IV, (5) end-stage incurable disease with reduced probability to survive the following 28 days, (6) pregnancy or breastfeeding, (7) age below 18 years, (8) *“Do Not Resuscitate”* (DNR) or *“Do Not Treat”* (DNT) order, (9) persistent vegetative stage (apallic syndrome), (10) participation in interventional studies and (11) familial relationship to member of study fellows.

This investigation and the experimental protocol were approved by the institutional ethics committee of the University of Goettingen in Goettingen, Germany. The study was performed in accordance with the provisions of the Declaration of Helsinki and in accordance with relevant guidelines and regulations. The methods were carried out in accordance with approved guidelines. Written informed consent was obtained either from the patient or their legal representative.

### Data collection

Clinical report forms (CRF) were created for all patients enrolled into the study. Upon enrollment, patients’ baseline characteristics including comorbidities, preexisting medication, and initial SOFA and APACHE II scores were obtained. Relevant parameters for organ-specific SOFA subscores were recorded daily for a maximum period of 28 days after sepsis onset, except patients who died or were dismissed from the ICU earlier. The clinical data were generated from the electronic patient record system (IntelliSpace Critical Care and Anesthesia (ICCA), Philips Healthcare, Andover, MA, USA). Parameters included fraction of inspired oxygen (FiO2) and arterial partial oxygen pressure (PaO2) for the respiratory system, mean arterial pressure (MAP) and quantity of vasopressor therapy for the cardiovascular system, Glasgow Coma Scale (GCS) for the central nervous system (CNS), bilirubin for the liver function, creatinine and urine output for the renal function and number of platelets for coagulation. Patients were followed up for 90 days, and mortality was assessed as a primary outcome variable.

### CTLA-4 rs231775 genotyping

DNA was automatically extracted from 200 µl of EDTA blood using a QIAamp® DNA Blood Kit in a QIAcube® or from 350 µl of EDTA blood using an EZ1® DNA Blood Kit in a BioRobot EZ1® or from peripheral blood mononuclear cells (PBMCs) using an AllPrep DNA Mini Kit according to the manufacturer’s instructions (all from Qiagen, Hilden, Germany). The quantity and quality of the DNA were determined by spectrophotometric measurement and revealed an average concentration of 33.8 ng/µl and an average E260/E280 extinction ratio of 1.85. Genotyping of the extracted DNA was performed via TaqMan polymerase chain reaction (PCR) using the predesigned TaqMan® SNP Genotyping Assay C___2415786_20 and a 7900HT Fast Real-Time PCR System (TaqMan) according to the manufacturer’s instructions (Life Technologies, Darmstadt, Germany). The genotyping outcomes were generated using 7900HT Fast Real-Time PCR System Software (SDS v2.4.1 for Windows 7, Applied Biosystems, Foster City, USA). For reliability reasons, 20% of the samples were genotyped in duplicate and showed completely concordant results. The DNA extraction and CTLA-4 rs231775 genotyping were performed entirely in the laboratories and under the supervision of the Department of Clinical Pharmacology of the University Medical Center Goettingen.

### Statistical analyses

STATISTICA 13 software (version 13.0, StatSoft, Tulsa, Oklahoma, USA) was used for statistical analyses and creation of the Kaplan-Meier diagrams. Probability values (p value) of p ≤ 0.05 were defined as “*significant*”, p ≤ 0.01 as “*very significant*” and p ≤ 0.001 as “*highly significant*”. The significance for categorical variables was calculated using the Pearson Chi-Square test or, if applicable, two-sided Fisher’s Exact test. The Mann-Whitney-U-test and Kruskal-Wallis test were performed for continuous variables. In the attached tables, continuous variables are presented as the mean ± standard deviation and categorical variables as absolute numbers or percentages. Time-to-event data were evaluated with the log-rank test of the Kaplan-Meier survival analysis. Multivariate Cox regression analysis was conducted to estimate the effect of multiple categorical and continuous independent variables on the time-to-event (e.g., survival) and calculation of their relative hazards. Observed allele frequencies in the study population were compared to an expected distribution of a normal population according to Hardy-Weinberg equilibrium and were examined using the chi-square test. A power analysis was performed with the appropriate Statistica analysis tools.

## Electronic supplementary material

Supplementary Dataset

## Data Availability

The datasets generated and/or analyzed during the current study are available from the corresponding author on reasonable request.
