# Supplementary material for: The CTLA-4 rs231775 GG genotype is associated with favorable 90-day survival in Caucasian patients with sepsis
Source: Sci Rep. 2018 Oct 11;8:15140. doi: 10.1038/s41598-018-33246-9 (PMC6181961; doi:10.1038/s41598-018-33246-9)

The CTLA-4 rs231775 GG genotype is associated with favorable 90-day survival in Caucasian patients with sepsis

Caspar Mewes, Benedikt Büttner, José Hinz, Ayelet Alpert-Jarbi, Aron Popov, Michael Ghadimi, Tim Beissbarth, Mladen Tzvetkov, Shai Shen-Orr, Ingo Bergmann, and Ashham Mansur

Supplementary Tables

Table 1: Baseline Characteristics with regard to CTLA-4 rs231775 genotypes

Parameter	All (n=644)	rs231775			p value
		GG (n=101)	AG (n=289)	AA (n=254)	
Age, years	63±15	63±14	62±15	65±15	0.0442
Male [%]	66	62	68	66	0.6003
Body Mass Index	28±6	29±7	27±6	28±6	0.0640
**Severity of sepsis**					
Septic shock, %	51	50	54	49	0.4040
Sequential Organ Failure Assessment (SOFA) score	9.4±3.9	9.8±3.9	9.6±3.9	9.0±3.9	0.1611
Acute Physiology and Chronic Health Evaluation (APACHE II) score	22±7	22±7	22±7	21±7	0.7524
**Comorbidities, n [%]**					
Hypertension	54	54	53	56	0.8339
History of myocardial infarction	5	2	5	7	0.1551
Chronic obstructive pulmonary disease	15	15	17	13	0.3102
Renal dysfunction	10	11	11	10	0.9306
Noninsulin-dependent diabetes mellitus	9	11	9	8	0.7252
Insulin-dependent diabetes mellitus	11	14	9	11	0.4448
Chronic liver disease	6	11	6	6	0.1278
History of cancer	16	13	17	15	0.5902
History of stroke	6	6	5	7	0.7596
**Recent surgical history, n [%]**					
Elective surgery	29	25	27	34	
Emergency Surgery	53	57	54	50	
No History of Surgery	18	18	19	16	
**Site of infection, n [%]**					
Lung	62	63	61	62	
Abdomen	20	22	18	22	
Bone or soft tissue	4	4	4	4	
Surgical wound	2	2	1	2	
Urogenital	2	1	4	2	
Primary bacteremia	7	3	8	7	
Other	3	5	4	1	
**Organ support [%]**					
Used during observation period					
Mechanical ventilation	93	95	92	94	0.6228
Use of vasopressor	79	81	81	76	0.2697
Renal replacement therapy	21	27	21	19	0.2265
Used on sepsis onset					
Mechanical ventilation	86	88	84	86	0.6313
Use of vasopressor	67	70	70	61	0.0781
Renal replacement therapy	9	14	8	8	0.1550
Use of Statins[%]	24	23	22	26	0.6767
ICU LOS	20±16	19±12	21±17	21±16	0.7807

Table 2: Disease severity with regard to CTLA-4 rs231775 genotypes

Variable	All (n=644)	rs231775			p value
		GG(n=101)	AG (n=289)	AA(n=254)	
SOFA	7.0±3.5	7.1±3.4	7.0±3.5	6.9±3.7	0.4693
SOFA-Respiratory score	2.0±0.8	2.0±0.7	2.0±0.8	1.9±0.8	0.8983
SOFA-Cardiovascular score	1.5±1.0	1.5±1.0	1.5±0.9	1.5±1.0	0.7934
SOFA-Central nervous system score	2.0±1.1	2.0±1.1	2.0±1.1	2.0±1.1	0.9877
SOFA-Renal Score	0.8±1.2	0.9±1.3	0.8±1.2	0.7±1.1	0.7615
SOFA-Coagulation score	0.4±0.6	0.3±0.6	0.4±0.6	0.4±0.6	0.7065
SOFA-Hepatic score	0.4±0.7	0.4±0.8	0.4±0.7	0.3±0.7	0.8659
Length of stay in ICU [days]	20±16	19±12	21±17	21±16	0.4538
**Organ support-free days**					
Ventilator-free days	5±5	5±5	4±5	5±6	0.4242
Vasopressor-free days	10±7	10±6	10±7	10±7	0.7626
Dialysis-free days	14±8	13±7	14±8	14±9	0.3110
Ventilation days/observation days, [%]	66±32	64±29	67±32	67±33	0.4206
Vasopressor days/observation days, [%]	34±30	34±30	34±29	33±32	0.5261
Dialysis days/observation days, [%]	9±23	12±23	10±23	8±22	0.2383
**Inflammatory values**					
Leucocytes [1000/µl]	13±5	13±4	13±5	13±5	0.6867
CRP [mg/l]	152±87	177±113	146±77	150±86	0.5478
Procalcitonin [ng/dl]	4.0±9.3	5.2±10.3	4.1±9.6	3.3±8.5	0.0828
**Kidney values**					
Urine output [ml/day]	2978±1337	2806±1179	2981±1368	3043±1360	0.5724
Urine output [ml/kg/day]	1.6±0.8	1.4±0.8	1.6±0.8	1.6±0.7	0.2179
Creatinine [mg/dl]	1.2±0.9	1.3±1.1	1.2±0.9	1.2±0.9	0.6383
**Liver values**					
AST (GOT) [IU/l]	169±598	162±329	167±708	173±537	0.3839
ALT (GPT) [IU/l]	94±188	90±174	88±151	101±228	0.7147
Bilirubin [mg/dl]	1.2±2.1	1.4±3.0	1.2±1.9	1.2±1.8	0.9012

Supplementary Figures

Figure 1: Kaplan Meier 90-day survival analysis with regard to CTLA-4 rs231775 genotypes

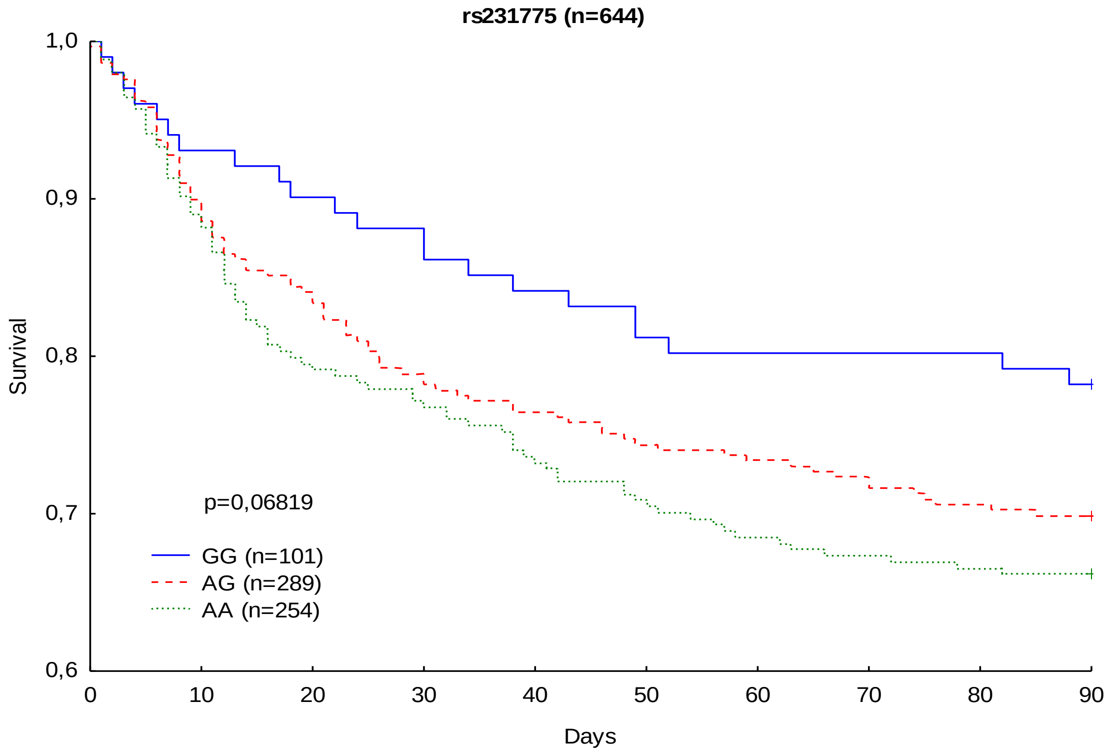

Figure 2: Kaplan Meier 28-day survival analysis with regard to CTLA-4 rs231775 genotypes

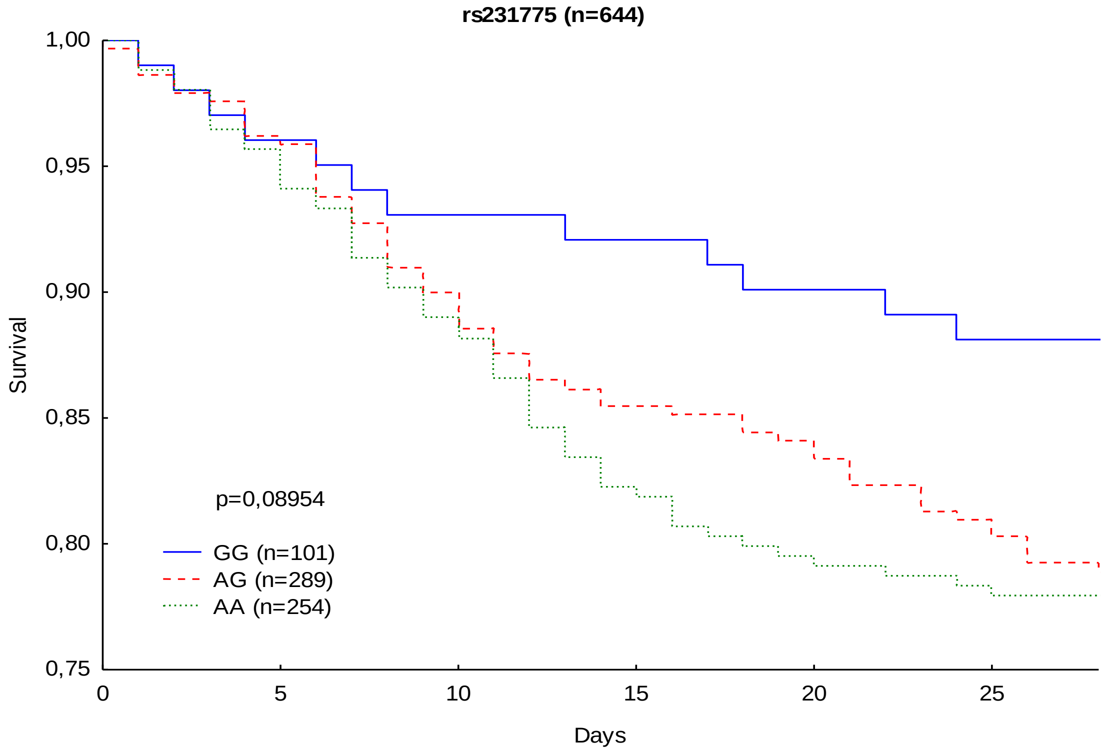

Supplement: Supplementary file 1 — Supplementary Dataset [file 41598_2018_33246_MOESM1_ESM.pdf]
